# Protocol for a randomised feasibility trial comparing a combined program of education and exercise versus general advice for ankle osteoarthritis

**DOI:** 10.1186/s13047-023-00669-1

**Published:** 2023-10-20

**Authors:** Michelle D. Smith, Viana Vuvan, Natalie J. Collins, David J. Hunter, Nathalia Costa, Melinda M. Franettovich Smith, Bill Vicenzino

**Affiliations:** 1 The University of Queensland, School of Health and Rehabilitation Sciences, Physiotherapy, Brisbane, QLD 4072 Australia; 2 La Trobe Sport and Exercise Medicine Research Centre, School of Allied Health, Human Services and Sport, La Trobe University, Melbourne, Australia; 3 Sydney Musculoskeletal Health, Kolling Institute, Rheumatology Department, The University of Sydney, Royal North Shore Hospital, Sydney, Australia; 4 Sydney School of Health Sciences, The University of Sydney, Physiotherapy, Sydney, NSW Australia; 5 School of Clinical Sciences, Queensland University of Technology, Brisbane, QLD Australia

**Keywords:** Ankle osteoarthritis, Feasibility, Exercise, Education

## Abstract

**Background:**

Ankle osteoarthritis (OA) is a serious problem with high associated pain and disability. While education and exercise are recommended for the initial management of OA, this has not been investigated in ankle OA. The primary aim of this study is to establish the feasibility of running a full-scale randomised controlled trial (RCT) investigating a combined education and exercise program compared to a general advice program for people with ankle OA. The secondary aims are to collect preliminary data which will inform sample size calculations, and understand the perspectives of people with ankle OA on their participation in the trial.

**Methods:**

Thirty individuals aged 35 years or older with symptomatic radiographic ankle OA will be recruited from the community and randomised to receive either a combined education and exercise program or a general advice program, both of which will be delivered by a physiotherapist in a group setting. Primary outcomes of feasibility include responses to study advertisements, number of eligible participants, recruitment rate, adherence with the intervention, fidelity of the intervention, adverse events, drop-out rate, and credibility and expectancy of the intervention. Secondary participant-reported outcomes will include global rating of change, patient acceptable symptom state, severity of ankle pain and stiffness, self-reported function, quality of life, satisfaction with treatment, and use of co-interventions. Follow up will be at 8 weeks and 3 months. Physical measures of 40 m walking speed, timed stairs descent, heel raise endurance and ankle dorsiflexion range of motion will be collected at baseline and 8 weeks. Primary feasibility outcomes will be reported descriptively, and estimates of the variability of secondary participant-reported and physical outcomes will be calculated. Semi-structured interviews will be conducted with participants to understand perspectives about the intervention and participation in the trial, with data analyzed thematically.

**Discussion:**

Study findings will establish the feasibility of running a full-scale RCT to investigate a combined education and exercise program compared to a general advice program for people with ankle OA. This study is a necessary first step to advance the international research agenda of evaluating the efficacy of exercise in the management of ankle OA.

**Trial registration:**

ACTRN12623000017628. Registered 10 January 2023, https://www.anzctr.org.au/ACTRN12623000017628.aspx.

**Supplementary Information:**

The online version contains supplementary material available at 10.1186/s13047-023-00669-1.

## Background

Ankle osteoarthritis (OA) is estimated to affect between 3.4% [1] and 6.5% [2] of the adult population and is a known long-term consequence of ankle sprains and fractures, which are among the most common injuries sustained in general and sporting populations [3]. Due to the post-traumatic nature, ankle OA affects individuals in their third decade of life [3]. People with ankle OA report “*crippling*” and “*limiting*” pain, which affects basic ambulation, participation in recreational activities, and work [4]. Quantitative research confirms these findings, with evidence of high levels of pain and disability, and low quality of life (QoL) [5]. Disability and QoL in ankle OA are similar to that reported by individuals with end-stage renal disease, radiculopathy and congestive heart failure [6].

There is little robust evidence to guide the management of ankle OA. A 2015 systematic review did not identify any evidence-based non-surgical interventions for ankle OA [7], and surgical management is prone to complications. It is estimated that 42% of ankle joint replacement surgeries require surgical revision [8], and 63% of patients experience adverse events [9]. With poor outcomes following surgery and the recognition that surgical management should be reserved for those who fail to respond to appropriate non-surgical management, there is an urgent need for effective, evidence-based non-surgical management for ankle OA.

Unlike hip and knee OA, there are no current clinical practice guidelines or recommendations for the management of ankle OA. Evidence-based guidelines for the management of hip and knee OA from the Osteoarthritis Research Society International (OARSI) state that all individuals should receive education about self-management and undertake regular aerobic and strengthening exercises [10]. The 2018 Royal Australian College of General Practitioners (RACGP) guidelines for the management of hip and knee OA recommend exercise and weight management as the first-line treatment [11]. Similarities in disease and reported impairments in pain, physical and emotional function in ankle [12, 13], knee [14] and hip [15, 16] OA suggest that similar approaches may be effective in managing these conditions. Physiotherapist-led exercise and education programs are associated with improvements in pain, function, QoL, physical activity, medication use, sick leave, and health care costs in people with hip and knee OA [17, 18]. However, the efficacy of such a program in ankle OA has not been investigated, and it is important not to extrapolate findings at the hip and knee to the ankle.

The primary aim of this study is to establish the feasibility of running a full-scale randomised controlled trial (RCT) investigating a combined education and exercise program compared to a general advice program for people with ankle OA. The secondary aims are to collect preliminary data to inform sample size calculations for a well-powered RCT, and understand the perspectives of people with ankle OA on their participation in the trial.

## Methods

### Study design

This feasibility study uses a mixed-methods approach with a central randomised parallel-group design and qualitative semi-structured interviews at trial completion. It follows the Standard Protocol Items: Recommendations for Interventional Trials (SPIRIT) guidelines [19], the CONSORT extension for randomised pilot and feasibility trials [20] and the Consensus on Exercise Reporting Template (CERT) [21]. Methodology and reporting of the semi-structured interviews will be guided by the Consolidated Criteria for Reporting Qualitative Studies (COREQ) [22]. The study is registered on the Australia New Zealand Clinical Trials Registry (ANZCTR; registration #: ACTRN12623000017628, registration date: 10 January 2023). Ethical approval has been obtained from The University of Queensland Human Research Ethics Committee (Approval #: 2018/HE002196) and all participants will provide informed consent prior to study participation.

### Participants

Individuals will be recruited using a broad recruitment strategy that has been successfully used in previous ankle OA research [13]. We will use paid and unpaid advertisements on social media (e.g., Facebook, Twitter), websites (e.g., Weekend Notes, Arthritis Australia, Arthritis Queensland), newsletters (e.g., UQ Update), radio (e.g., 4BC), and electronic and physical noticeboards in the greater Brisbane area. Posters will be given to physiotherapy and inter-professional health clinics to post in their practices. Advertisements will use simple language and include a range of images to appeal to diverse individuals.

Formal sample size calculations are not applicable for feasibility studies. We chose a sample of 30 participants for feasibility of recruitment within the 1-year study period, while allowing observation of sample variability and any adverse responses. Individuals with ankle OA will be required to meet the following eligibility criteria for inclusion: (i) aged over 35 years; (ii) ankle joint pain on most days for the last three months; (iii) severity of ankle pain in the last week ≥ 3 out of 10 on an 11-point numerical rating scale (NRS) anchored with ‘no pain’ at 0 and ‘worst pain imaginable’ at 10; (iv) modified Kellgren & Lawrence scale (K&L) OA grade ≥ 2 at the subtalar and/or talocrural joints defined as the presence of osteophytes and/or joint space narrowing [23]; (v) committed to undergo the allocated treatment and undertake all follow up outcome measurements; and (vi) able to understand verbal and written English. Study exclusion criteria are: (i) health problems or pain elsewhere that are more concerning than that at the ankle; (ii) received exercise and/or education-based treatment for ankle OA in the last three months; (iii) previous ankle arthrodesis or total ankle replacement on the affected ankle; (iv) neurological, vestibular or systemic arthritic conditions; (v) receiving treatment for cancer; and (vi) inability to undertake radiographic evaluations (e.g. pregnancy) and/or participate in the treatment program.

### Study procedures

**Fig. 1:**
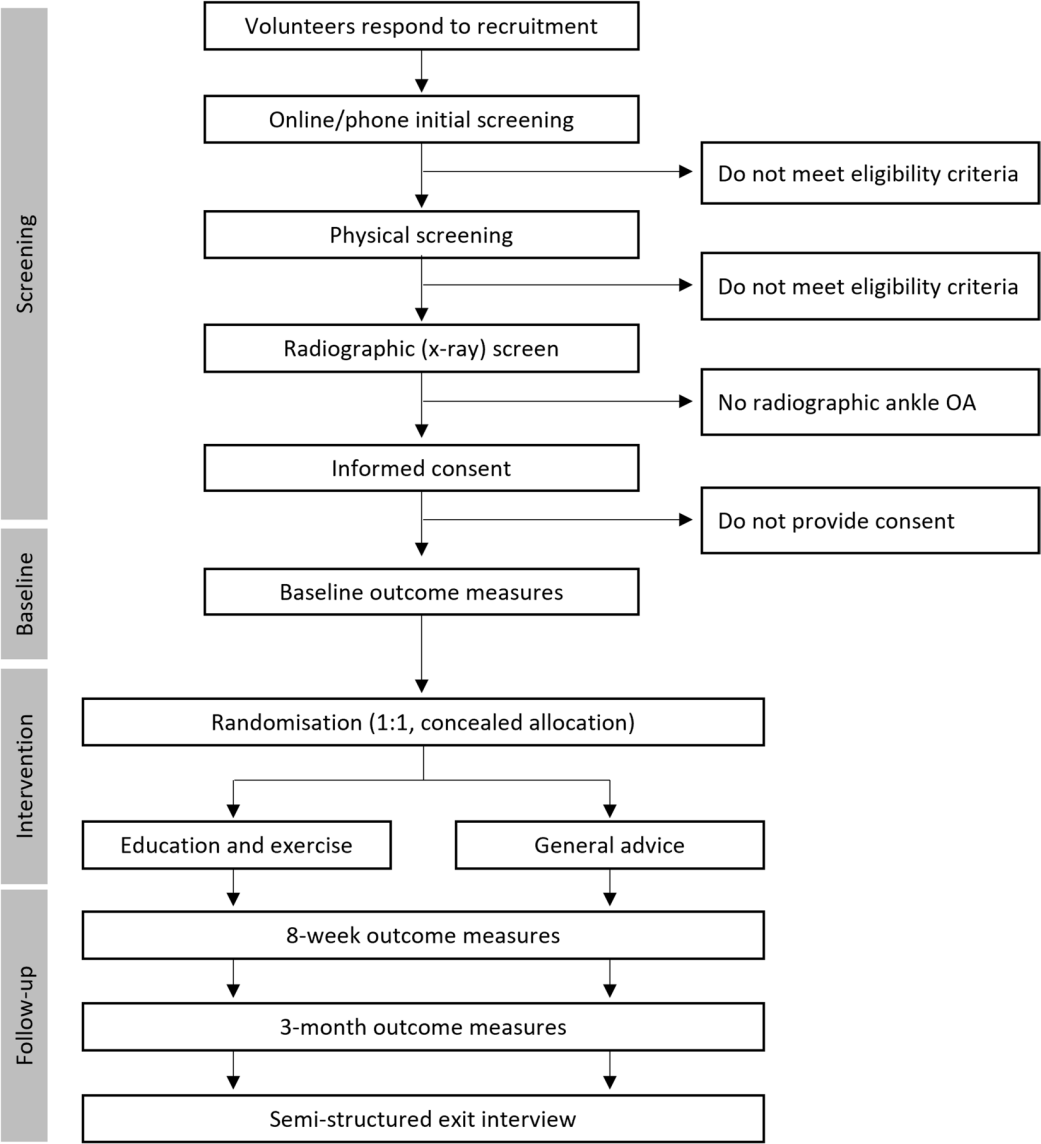
Participant flow through the trial

The flow of participants through the study is shown in Fig. 1. Individuals who express interest in participating in the trial will complete a multi-stage screening process. First, they will complete a preliminary online screening survey and verbal phone screen to check eligibility. Second, individuals will undertake a comprehensive in-person physical examination by a registered physiotherapist to ensure that the individual’s ankle pain is arising from the ankle joint and not surrounding structures. Finally, individuals will undergo lateral and mortise view ankle x-rays at a radiology clinic to determine the presence of radiographic ankle OA at the subtalar and/or talocrural joints. Radiographs will be assessed by a researcher with radiology training to determine the stage of OA [23]. Interrater reliability of grading radiographic ankle OA severity in previous research from our group is substantial (Kappa: 0.69 (95% confidence intervals: 0.59, 0.79) [13]. Individuals who meet all eligibility criteria will complete informed consent documentation and baseline data collection with a research team member blinded to group allocation.

Study participants will be randomised using 1:1, concealed allocation to receive either: (a) physiotherapist-delivered education and exercise; or (b) physiotherapist-delivered general advice. The randomisation schedule will be prepared by an investigator independent to participant screening and data collection (NJC). The randomisation sequence will be generated using a random number generator with random blocks (blocks 2–6). To conceal randomization, consecutively numbered, sealed, opaque envelopes will be used and stored in a locked location. An unblinded member of the research team (VV) will reveal the participant’s intervention allocation.

Due to the nature of the interventions being assessed in this trial, it is not possible for participants or physiotherapists delivering the intervention to be blinded to the treatment delivered. The primary feasibility outcomes will be calculated and analysed by a blinded assessor (MDS). Credibility, expectancy and secondary participant-reported outcomes will be reported by study participants who cannot be blinded to the intervention received. While participants (assessors of self-report measures) will be aware of the two treatments being compared, study hypotheses will not be disclosed. Assessor evaluated outcomes will be undertaken by an assessor who is blinded to participant treatment allocation.

### Interventions

Trial interventions will be delivered by private practice physiotherapists who are registered with the Australian Health Practitioner Regulatory Agency and have at least five years of clinical experience and post-graduate physiotherapy qualifications. To ensure consistency in the delivery of interventions, trial physiotherapists will attend a training session on the trial methods and intervention protocols. Study interventions will be offered at two private practice physiotherapy clinics in different areas of Brisbane. Participants will select the physiotherapy clinic that is most convenient for them to attend.

#### Education and exercise intervention

The combined education and exercise intervention will be delivered in a supervised class format with 2–4 participants per class. Consistent with OARSI guidelines [10], participants will be provided education about OA, diagnosis and symptoms, international guidelines and evidence-based management. This information will be delivered in two group education sessions in the first week (week 1) of the intervention. The education session will be facilitated by a PowerPoint presentation and will contain ample opportunity for discussion and questions. Participants will be provided a handout of the PowerPoint presentation.

The exercise sessions follow resistance training guidelines from the American College of Sports Medicine [24]. There will be two group exercise sessions per week over six weeks (a total of 12 exercise sessions, each of 1-hour duration) delivered by a trial physiotherapist (weeks 2–7 of the intervention). There will be 2–3 days of rest between exercise sessions. The exercise program will include aerobic exercise (i.e., stationary bike), exercises for foot/ankle muscle strength (i.e., resistance band and body weight exercises), compound lower limb exercises (i.e., step ups), postural control/balance training (i.e., standing on compliant surfaces) and gentle stretching (Table 1). Strengthening exercise sessions will be prescribed as two sets of 10–12 repetitions and 1 min rest between sets. Load (Theraband® and free weight resistance) will be individualised to study participants and will be an intensity that the participants can perform between a maximum of 10 and 12 repetitions with correct form/technique and a patient-acceptable level of pain (determined dichotomously with the question ‘Are your current symptoms acceptable, when you take your general functioning and your current pain into consideration?’). If the participant cannot perform 10 repetitions with correct technique or acceptable pain, a lighter load/resistance will be used. If the participant can perform more than 12 repetitions with good technique and acceptable pain, then a heavier load/resistance will be used. The load/resistance will be individually evaluated by the physiotherapist for each exercise and load will be progressed to maintain this workload. Each exercise repetition will consist of a 2-second concentric phase, 1-second isometric hold, and 2-second eccentric phase with a 1-second rest between repetitions. Each set will take about 60–70 s to complete with a total time under tension of 50–60 s per set (and 100–120 s per exercise). Exercises done unilaterally will be performed on both legs, with one set of the exercise performed on the unaffected side. If time does not allow performance of all exercises bilaterally, the resisted foot and ankle exercises will be omitted on the unaffected side. The detailed exercise program, including prescription and progressions, is outlined in Supplementary file 1.

**Table 1:** The components of the exercise program

**Warm up/ Aerobic exercise**	10 min of cycling on a cycle ergometer at a workload of ‘somewhat hard’ on the rate of perceived exertion (RPE) scale [25].
**Foot and ankle specific muscle strength (5 exercises)**	Five exercises for foot and ankle-specific muscle strength using body weight resistance or Theraband® resistance. The specific exercises are: calf raises (body weight resistance) and Theraband® resisted ankle dorsiflexion, ankle inversion, ankle eversion, and toe flexion.
**Compound lower limb muscle exercises (3 exercises)**	Three compound lower limb muscle strength exercises which include step up/downs (body weight resistance), squats/resisted knee extension (body weight or Theraband® resisted), and a pelvic lift/supine bridge (body weight resistance).
**Balance training (1 exercise)**	A standing balance exercise series with adjustment to base of support and addition of perturbations.
**Cool down**	Walking forward and backwards for 2–3 min at a self-determined comfortable pace, followed by gentle stretching of the ankle plantarflexors, quadriceps and hamstrings. Stretches are held for 30 s each.

Physiotherapists will record attendance; intensity (e.g., Theraband® colour or kilogram load), repetitions and sets for each exercise; patient-acceptable level of pain (acceptable vs. not acceptable); and any adverse events for each exercise session. If participants report an unacceptable level of symptoms following the previous exercise session, load will be carefully monitored and adjusted as needed.

Participants will not be given a home exercise program during the six weeks of group exercise classes. At the completion of the 6-week program, participants will be instructed to continue with the exercises twice a week at home (i.e., a home exercise program). A handout will be provided with instructions on how to complete each exercise, and when and how to progress.

#### General advice intervention

Participants allocated to the general advice group will attend one 1-hour group session, where they will receive general information about OA (e.g., the importance of keeping active and tips on managing symptoms) based on online resources from Musculoskeletal Australia (*Understanding Osteoarthritis*) [26] and Arthritis Australia (*Information Sheet on Physical Activity*) [27]. They will be provided with the online resources in advance of their group session. The group session will be guided by a PowerPoint presentation to facilitate discussion about the information in the resources, and ample opportunity will be provided for participants to ask questions. Participants will receive a half-page handout of tips reinforcing the advice provided in the presentation and the contact details for the physiotherapist. No manual treatment or exercise prescription will be provided. Physiotherapists will record attendance at the general advice session. This is a similar treatment to other RCTs on musculoskeletal conditions and has been found to be acceptable to participants (based on recruitment rates, retention and participant feedback) [28].

#### Concurrent treatments

Participants will be requested to refrain from seeking other treatments during the trial, and report any interventions used. If participants use regular medication (e.g., analgesic and anti-inflammatory drugs), orthoses, prescribed footwear or assistive devices (e.g., walking aids) on entry to the study, they will be permitted to continue with their use.

### Outcome assessment

Baseline participant demographic (e.g., sex, weight, height) and ankle OA (e.g., affected ankle, aggravating activities, and modified Kellgren & Lawrence scale (K&L) OA grade [23]) data will be collected. Participant-reported outcome measures will be collected at baseline, 8 weeks and 3 months post-commencement of the intervention using an online survey platform (Qualtrics). Follow up data will be collected on all participants, including those who deviate from or discontinue their treatment allocation.

Participants will complete weekly diaries throughout the 3-month study period to record any adverse events in relation to their ankle or general health, any treatment they sought and any changes to medication they take for their ankle. Diaries will be given to participants at their first treatment session, with instructions to email a photo of the diary to their physiotherapist weekly (or to return it at the exercise session for those in the combined education and exercise intervention). Participants will receive a weekly reminder from their physiotherapist to submit the diary. Use of interventions outside of the study will be recorded as type (e.g., medication use, physiotherapy), number and percentage.

#### Primary outcomes

The primary outcome measures for this trial will be the feasibility of conducting a full-scale RCT to investigate a combined education and exercise program in the management of ankle OA. Feasibility will be evaluated using the following outcomes which will be assessed at the conclusion of the study:

- Individuals’ interest in the study (number of responses to study advertisements);
- Number (percentage) of eligible individuals from interested individuals;
- Recruitment rate (average number of study participants enrolled per month);
- Consent rate (percentage of consented participants from eligible individuals);
- Participant adherence with allocated intervention (the number (percentage) of sessions attended and reasons for absence);
- Physiotherapist fidelity delivering the intervention, evaluated by observation by a researcher not involved in intervention delivery using the fidelity assessment from Davis et al. [29];
- Adverse events (number and type) recorded by the physiotherapist at each exercise session and from participant diaries;
- Completion rate (number (percentage) of participants who do complete the intervention and 8-week and 3-month outcome measures) and reasons for non-completion/drop out.

Based on previous feasibility studies [30] and exercise adherence in knee OA research [31] (in light of no previous studies investigating or reporting adherence in ankle OA), the criteria for determining feasibility of conducting a full-scale RCT using the current protocol are reported in Table 2.

**Table 2:** Feasibility criteria for a future RCT

Outcome	Proceed	Proceed with caution	Do not proceed
Consent rate	> 50%	30–50%	< 30%
Participant adherence	> 60%	30–60%	< 30%
Intervention fidelity	> 60%	30–60%	< 30%
Completion rate of PROM ^a^ at 3 months	> 70%	50–70%	< 50%

*PROM *Participant-reported outcome measures

^a^Completion of at least severity of ankle pain and Global Rating of Change

To inform feasibility, credibility (how believable/logical the treatment is) and expectancy (expectations from treatment) will be evaluated using the Credibility/Expectancy Questionnaire [32]. This questionnaire has high internal consistency and test-retest reliability [32]. It will be completed at baseline, 1-week post-treatment commencement (after the first week of treatment has been completed), and 8-weeks post-treatment commencement (after the final treatment session for the education and exercise group).

#### Secondary outcomes

The secondary outcomes will be used to calculate variability in measures. The participant-reported outcome measures will be collected at baseline, 8 weeks and 3 months post-treatment commencement (except the G*lobal Rating of Change* and *Participant satisfaction with treatment* which will be assessed at 8 weeks and 3 months only*).*

*Severity of ankle pain* and *stiffness* (worst and average pain/stiffness in the last week) will be evaluated using 11-point NRS (0 = no pain/stiffness, 10 = worst pain/stiffness imaginable). The NRS has established reliability and validity in musculoskeletal research [33].

G*lobal Rating of Change* will be measured by asking the participant to indicate the overall change in their ankle condition on a 7-point Likert scale (answer options: Much better, Better, Slightly better, Same, Slightly worse, Worse, Much worse). This scale has been shown to be stable and clinically relevant for interpreting participant-perceived improvements in their condition [33].

*Patient Acceptable Symptom State*, which is the “highest level of symptoms beyond which participants consider themselves well”, will assess overall acceptability of the condition from the perspective of the participant [34]. Participants will be asked to answer Yes or No to the question: ‘Considering all the activities that you do in your daily life, how well you can do these activities, and your level of pain, do you think that your current state is satisfactory?’.

Self-reported function will be assessed using the 21-item *Activities of Daily Living sub-scale* and the 8-item *Sport sub-scale* of the *Foot and Ankle Ability Measure (FAAM)* [35]. Items are rated on a Likert scale and summed for a total score, represented as a percentage. The FAAM has excellent test-retest reliability and internal consistency [35]. Function measured with the FAAM has been shown to be impaired in individuals with ankle OA [5].

The *Foot and Ankle Outcome Score (FAOS)* is a self-report measure of ankle pain, symptoms, function and quality of life [36]. The FAOS is a valid and reliable instrument for assessing outcomes following ankle fractures [37] and ankle ligament reconstruction [36].

The *Patient Specific Functional Scale (PSFS)* will measure self-reported function in relation to functional tasks that the participant has difficulty with [38]. The participant will nominate up to 5 functional tasks and rate each task on a scale from 0 to 10 (0 = unable to do the task, 10 = able to do unimpeded with no symptoms).

Health-related quality of life will be measured using the EuroQol Group *EQ-5D* [39]. This questionnaire measures health-related quality of life in relation to five dimensions - mobility, self-care, usual activity, pain/discomfort and anxiety/depression.

*Participant satisfaction* with treatment will be assessed on a 5-point Likert scale (answer options: Very satisfied, Somewhat satisfied, Neither satisfied or dissatisfied, Dissatisfied, Very dissatisfied).

Four physical outcomes will be collected at baseline and 8 weeks post-treatment commencement. For each outcome, the best result obtained at each timepoint will be used for analysis.

The *timed 40 m fast-paced walk test* will measure ambulatory function [40]. The time, recorded with a stopwatch, to walk a 40 m distance over 4 × 10 m lengths (with turning time excluded) will be recorded. Walking speed will be calculated by dividing distance (40 m) by time. The use of a regular walking aid is permitted. This performance-based test is recommended by OARSI to assess physical function in people with hip and knee OA [40], and has excellent reliability [41]. This test will be performed twice with a 30-second rest between repetitions.

*Time to descend 1 flight of 10 stairs* will be recorded to assess physical function [13]. Participants will be instructed to descend the stairs as quick as possible. The time will be recorded with a stopwatch. This test has been shown to be reliable and impaired in individuals with ankle OA compared to controls [13]. This test will be performed twice with a 30-second rest between repetitions.

*Number of heel raises until fatigue* will measure calf capacity [13, 42]. Participants will stand on flat ground facing a wall, with their fingertips resting on the wall for balance support. Standing on one leg (their test leg), they will lift their heel as high off the ground as possible repeatedly in pace to a metronome set to 80 bpm (2 beats up and 2 beats down) until fatigue. The test will be stopped when the participant is unable to perform any more repetitions or when they cannot keep pace with the metronome, the height of the heel raise diminishes, excessive weight is placed through the hands, or the knee flexes. This test has excellent reliability [42] and has been shown to identify impaired calf capacity in individuals with ankle OA compared to controls [13]. This test will be performed once on each leg.

Dorsiflexion range of motion will be measured using the *knee to wall test* [43]. The participant will lunge their knee forward as far as possible to dorsiflex the ankle while keeping the foot perpendicular to the wall, knee over the second toe and the heel in contact with the ground. The distance between the forward projection of the knee and the longest toe will be measured with a ruler. The knee to wall test is a measure of talocrural joint dorsiflexion [43] and has excellent reliability [44]. Participants will be given two practice trials followed by three trials for data collection, with a 30-second rest between repetitions.

#### Qualitative data

Semi-structured interviews will be conducted at the conclusion of the study (within two weeks of the 3-month follow-up appointment) to understand participants’ perspectives about their allocated intervention and participation in the trial to inform the development of a full-scale randomised controlled trial (RCT) investigating a combined education and exercise program compared to a general advice program for people with ankle OA. Interviews will follow a semi-structured interview guide that will ask participants about what they liked and did not like about the trial, barriers and facilitators to participating in the trial, and their feelings about the intervention they were allocated. All participants will be invited to participate in an interview which will be conducted by an investigator not involved in the delivery of the intervention. Interviews will be audio recorded and transcribed verbatim using a professional transcription service.

### Data management

Online survey data will be downloaded to Microsoft Excel, and data for physical outcome measures will be entered into Microsoft Excel (via double data entry) and checked for accuracy/errors. All outcome measure data will be de-identified by the research assistant using individual participant codes. Treatment allocation will be added to the spreadsheet using a code that is not known to the researcher undertaking the data analysis, to ensure that they are blinded to group allocation. Physiotherapy notes (including attendance/adherence to the interventions) and participant diaries and adverse events will be entered into a separate Microsoft Excel spreadsheet with the participant ID and coded treatment allocation. Data will be stored on a on a password-protected server, only accessible by the research team.

### Data and statistical analysis

We will analyse and present data pertaining to the primary outcome measures descriptively. Estimates of the variability of participant-reported and physical outcomes (e.g., mean (standard deviation) for normally distributed data; median (inter-quartile range) for non-normally distributed data) will be calculated separately for the combined education plus exercise group and for the general advice group.

The perceptions of participants recorded at exit interviews will undergo thematic analysis [45]. Interview transcripts will be checked for accuracy in relation to the interview recordings. We will use a reflexive thematic approach to analyse the data and follow the steps described by Braun and Clarke [45]. While this analytical approach is not a homogenous procedure, our analysis will involve familiarisation with the data through reading the transcripts and taking notes, manually coding each interview to generate codes, grouping codes into initial themes and subthemes, and refinement of codes, themes and subthemes, which will occur through review and discussion with the authorship team throughout the analysis process. Rigour will be guided by Braun and Clarke’s recommendations for reflexive thematic analysis, such as inclusion of a justification of how reflexive thematic analysis is consistent with the research aims, discussion of the theoretical underpinnings of the research, and reflexivity (critical reflection on the researchers’ role in the research practice and process) [46].

### Dissemination of results

Study results will be published in peer-review journals and presented at national and international conferences. Key findings will be communicated to participants in lay terms following the completion of the trial.

## Discussion

Ankle OA is a serious problem that is associated with high disability, low self-reported function and low quality of life [5]. The symptoms and impairments that accompany ankle OA impair mobility, which limits participation in recreational, occupational and sporting physical activity, causes people to withdraw from social activities, and, in turn, compromises ability to “*enjoy life*” [4]. These findings highlight the broad impacts of ankle OA on an individual and society, and the importance of finding interventions that improve symptoms, participation and quality of life.

The International Foot and Ankle Osteoarthritis Consortium, an international group of expert foot and ankle clinicians and researchers associated with OARSI, developed a preliminary research agenda for foot and ankle OA research based on the identification of gaps in evidence and perspectives of clinicians and researchers [47]. One of the treatment-focused research agenda items is to “evaluate the efficacy of exercise in the treatment of foot and ankle OA”. Our study is an initial and necessary step to ascertain the efficacy of exercise, combined with education – an essential intervention for people with musculoskeletal pain [48] – in the treatment of ankle OA. Findings will establish feasibility and inform sample size calculations for a full-scale RCT.

Minimal research has investigated the use of exercise in the management of ankle OA, with only one study investigating exercise as a stand-alone intervention. Karatosun et al. 2008 [49] compared a 6-week home exercise program with three injections of hyaluronic acid. The exercise program consisted of isometric exercises for the ankle plantarflexors, dorsiflexors, invertors and evertors (exercise load/intensity not stated); stretching and active ankle range of motion; and strengthening of the foot intrinsic and quadriceps muscles, closed kinetic chain exercises and proprioceptive exercises (none of which were described). Pain severity, activity limitations and walking distance improved in both the hyaluronic acid and exercise groups 12 months post-treatment compared to baseline, with no differences between treatment groups. Two studies have included exercise in the investigation of combined interventions. Qi et al. [50] compared a combined intervention of a corticosteroid injection, massotherapy and exercise (which was not described) with a corticosteroid injection alone. They reported greater improvements in pain, swelling, dysfunction and quality of life at 1, 2 and 4 weeks post-treatment in the combined intervention group compared to the corticosteroid injection group. Finally, Sun et al. [51] compared a combined intra-articular hyaluronate injection and exercise intervention with an intraarticular botulinum toxin A injection. The exercise program was described as calf stretching, active ankle range of motion, balance board exercise and isometric ankle plantarflexion, dorsiflexion, inversion and eversion performed three times per week for four weeks (with no description of load). The authors reported improvements in pain and disability 6 months post-treatment, but no difference between groups.

While these exercise interventions appear to target ankle muscle strength, range of motion and postural control/balance, the lack of reporting of the specific exercises and prescription parameters (e.g., load, repetitions) makes appraisal and implementation of the exercise program difficult [52]. In light of deficits in ankle muscle strength and endurance [12, 13], range of motion [12, 13], postural control/balance [53, 54] and ambulatory function [13] present in individuals with ankle OA, it is hypothesized that appropriately prescribed exercises may address deficits, and in turn improve mobility, function, participation and QoL. The exercise program in our trial specifically targets impairments in ankle OA that have been identified in case-control studies, and includes exercises to improve proximal muscle function (e.g., squats and pelvic lift/supine bridge) which is impaired in people with chronic ankle problems [55]. For completeness of reporting and to facilitate implementation, the interventions are described in accordance with CERT guidelines [21], the Template for Intervention Description and Replication (TIDieR) checklist [52], and the exercise program is described based on guidelines from Toigo et al. [56].

The exercise intervention in our trial is combined with education, which is recommended for managing OA [11, 57, 58] and musculoskeletal pain conditions [48]. The education included in the combined exercise and education intervention is consistent with international guidelines for the treatment of OA. It includes education about the etiology and diagnosis of OA [11, 57, 58], risk factors [11], treatment options (including supporting evidence, benefits and harms) [11, 57, 58], self-management (including pacing of activities) [10, 57], exercise and physical activity recommendations and misconceptions [10, 11, 57], and the importance of maintaining a healthy body weight [10, 11, 57, 58].

The comparator intervention in this trial is general advice, provided by a physiotherapist in a group setting (to have patient-therapist interaction, psychosocial contact and interaction with other individuals in both interventions [28]). The general advice intervention is similar to comparator/control interventions used in previous RCTs in which participants received an information session with a physiotherapist accompanied by a handout about the condition, strategies for self-management and the benefits of physical activity [28, 59]. The use of information from freely available online resources from national organizations (i.e., Musculoskeletal Australia and Arthritis Australia) for the general advice intervention is consistent with that used in previous research [28].

Group interventions were chosen for this trial due to the benefits of social interaction between class participants and the economics of physiotherapist time and resources [60]. Physiotherapists delivering group exercise interventions for patients with hip and knee OA believe that the group environment improves program attendance, increases interactions and questions from patients, and facilitates the creation of a supportive environment [61]. A systematic review and meta-analysis did not find any clinically significant differences in pain and disability in the short-term between group and one-on-one physiotherapist-delivered exercise interventions for individuals with musculoskeletal conditions, but group exercise interventions were slightly more effective in the medium and long-term [62].

This study follows the Standard Protocol Items: Recommendations for Interventional Trials (SPIRIT) guidelines [19] and the CONSORT extension for randomised pilot and feasibility trials [20], and was prospectively registered on the Australia New Zealand Clinical Trials Registry (ANZCTR; registration #: ACTRN12623000017628). There are several strengths in the study design. Participants will be randomised to interventions, allocation to interventions will be concealed, and researchers undertaking assessment of outcome measures and analyses of data will be blinded to participant group. The educational content for the combined exercise and education group and the general advice content for the general advice group were reviewed by an individual with ankle OA prior to study commencement, and feedback was used to make adjustments to content and delivery. The outcome measures in the trial were chosen to align with a recently established core domain set for ankle OA, which is a minimum set of domains that should be measured in all ankle OA research to adequately measure the impacts of the condition [63, 64]. The five domains included in the core domain set for ankle OA are pain severity, health-related quality of life, function, disability and range of motion [65].

This trial will establish the feasibility of conducting a full-scale RCT investigating a combined education and exercise program compared to a general advice program for people with ankle OA. We have nominated four feasibility criteria to guide the decision of whether or not to proceed to an RCT using the current protocol – consent rate, participant adherence, intervention fidelity and completion rate of participant-reported outcome measures at three months. Ankle OA research that has included an exercise component in the intervention has not reported consent rates, adherence or fidelity [49–51]. Thus, we have based our decisions for these criteria on a feasibility study by Bateman et al. [30], and reports of ≥ 60% weekly adherence to a 12-week exercise program in people with knee OA [31]. As there are no core outcome sets for ankle OA (specific outcome measures recommended to assess core domains) [65], we have included a range of participant-reported outcome measures with an aim to determine those with best utility in a RCT. We have based our completion rate of these measures on a minimal set of outcomes – the single item scales of severity of ankle pain and Global Rating of Change, which have been used as primary outcomes in many RCTs on musculoskeletal conditions [28, 59].

## Conclusion

The effectiveness and importance of non-surgical management are increasingly being recognized in OA, and clinical guidelines reinforce the need for the provision of appropriate non-surgical management before surgical intervention is considered. This study will establish the feasibility of running a full-scale RCT to investigate a combined education and exercise program compared to a general advice program for individuals with ankle OA. Secondary aims will enable the calculation of variability in outcomes to inform sample size calculations for a well-powered RCT. The embedded qualitative interviews will provide key insights from participants on their perspectives on the intervention to inform future trials. This study is an important step to advance evidence-based care for people with ankle OA.

### Supplementary Information

**Additional file 1.**

## Data Availability

The datasets generated and/or analysed during the feasibility study will be available in The University of Queensland eSpace and available from the corresponding author on reasonable request.
