# Supplementary material for: Protocol for a randomised feasibility trial comparing a combined program of education and exercise versus general advice for ankle osteoarthritis
Source: J Foot Ankle Res. 2023 Oct 20;16:72. doi: 10.1186/s13047-023-00669-1 (PMC10588035; doi:10.1186/s13047-023-00669-1)
Supplement: Supplementary file 1 — Additional file 1. [file 13047_2023_669_MOESM1_ESM.pdf]

Supplementary File 1. Description of neuromuscular exercises in the combined exercise and education intervention.

Exercise	Description	Load	Reps	Sets	Speed	Rest	TUT	Level 1	Level 2	Level 3	Level 4
**1**<br>Calf raises<br>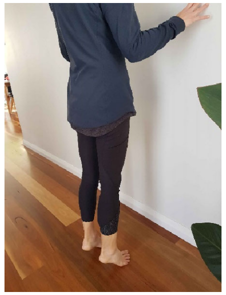	In standing, raise onto your toes as high as you can by lifting your heels off the ground. Slowly lower your heels back to the ground. (If not able to perform, perform seated calf raises with a weight across the thighs as able.)	~12 RM	10-12 reps	2 sets	2 s concentric<br>1 s isometric<br>2 s eccentric	1 s between reps;<br>1 min between sets	50-60s/ set	Stand feet hip width apart, equal weight on both legs and light hand support for balance.	Stand with feet hip width apart, equal weight on both legs and light hand support for balance. Hold a hand weight (increase load as needed).	Stand on one leg and slight hand support for balance.	Stand on one leg and light hand support for balance. Hold a hand weight (load for 12RM).
**2**<br>Resisted ankle dorsiflexion<br>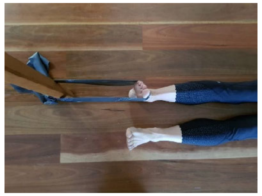	Sit in long sitting, back supported and a band resisting the <u>top</u> of your foot. Bend your foot towards your face as far as you can to pull the band. Slowly return to the starting position.	~12 RM	10-12 reps	2 sets	2 s concentric<br>1 s isometric<br>2 s eccentric	1 s between reps;<br>1 min between sets	50-60s/ set	Isometric dorsiflexion against Theraband resistance with maximal tolerated load (5 sec hold x 6 reps x 2 sets).	Use a light resistance band for strengthening through range of motion (yellow or red).	Use a medium resistance band for strengthening through range of motion (green or blue).	Use a heavy resistance band for strengthening through range of motion (black or silver).
**3**<br>Resisted ankle inversion<br>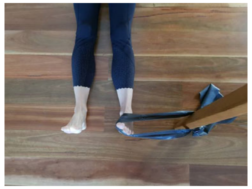	Sit in long sitting, back supported and a band resisting the <u>inside</u> of your foot. Turn your foot inwards towards your opposite leg as far as you can to pull the band. Slowly return to the start position.	~12 RM	10-12 reps	2 sets	2 s concentric<br>1 s isometric<br>2 s eccentric	1 s between reps;<br>1 min between sets	50-60s/ set	Isometric inversion against Theraband resistance with maximal tolerated load (5 sec hold x 6 reps x 2 sets)	Use a light resistance band for strengthening through range of motion (yellow or red).	Use a medium resistance band for strengthening through range of motion (green or blue).	Use a heavy resistance band for strengthening through range of motion (black or silver).

**4**<br>Resisted ankle eversion<br>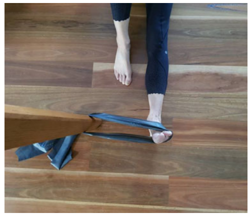	Sit in long sitting and back supported and a band resisting the <u>outside</u> of your foot. Turn your foot outwards as far as you can to pull the band. Slowly return it to the starting position.	~12 RM	10-12 reps	2 sets	2 s concentric<br>1 s isometric<br>2 s eccentric	1 s between reps;<br>1 min between sets	50-60s/ set	Isometric eversion against Theraband resistance with maximal tolerated load (5 sec hold x 6 reps x 2 sets)	Use a light resistance band for strengthening through range of motion (yellow or red).	Use a medium resistance band for strengthening through range of motion (green or blue).	Use a heavy resistance band for strengthening through range of motion (black or silver).
**5**<br>Resisted toe flexion<br>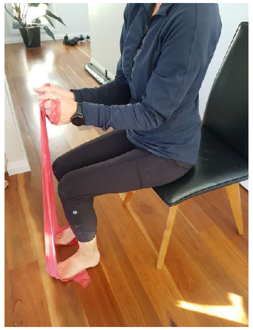	Sit/stand with your feet hip width apart. Place the ends of a resistance band flat under each foot and hold the middle of the band in your hands. Pull upward on the band, allowing the band to extend your toes off the ground as far as you can. Keep the toes straight and slowly push them into the band until they reach the floor. Slowly control the return back up.	~12 RM	10-12 reps	2 sets	2 s concentric<br>1 s isometric<br>2 s eccentric	1 s between reps;<br>1 min between sets	50-60s/ set	Sitting in a chair. Use a light resistance band for strengthening through range of motion (yellow or red).	Standing with your hands on your thighs. Use a light resistance band for strengthening through range of motion (yellow or red).	Standing as per Level 2. After you push your toes to the floor, hold and slide your hands down your thighs while you perform a squat. Return to standing and control the lift of your toes off the ground. Use a light resistance band for strengthening through range of motion (yellow or red).	Repeat Level 3 with a medium resistance band (green or blue).<br>Notes: Perform Level 2 with a medium resistance band if Level 3 is too difficult.

**6A**<br>Squats<br>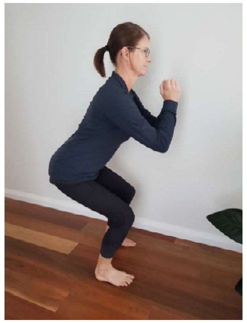<br>**6B**<br>Resisted knee extension (if unable to do 6A)	**6A**: Stand with feet hip width apart, squat down to 60° of knee flexion by sitting backwards. Keep your knee positioned above your 2nd toe. Slowly raise to the starting position.<br>**6B**: Sit in 90° of knee flexion with a band around the front of your foot/ankle. Straighten your knee as far as you can to pull the band. Slowly bend your knee to return to the starting position.	~12 RM	10-12 reps	2 sets	2 s concentric<br>1 s isometric<br>2 s eccentric	1 s between reps;<br>1 min between sets	50-60s/ set	**6A:** Squat to approximately 60 degrees (or less if needed) with no load.<br>**6B**: Use a light resistance band (yellow or red).	**6A:** Squat to approximately 60 degrees with light load (hand weights).<br>**6B**: Use a medium resistance band (green or blue).	**6A:** Squat to approximately 60 degrees with moderate load (hand weights).<br>**6B**: Use a heavy resistance band (black).	**6A:** Squat to approximately 60 degrees with heavy load (hand weights).<br>**6B**: Use a very heavy resistance band (silver).
**7**<br>Step up/step downs<br>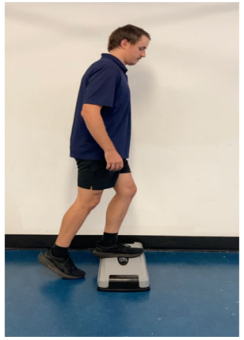	Step up onto a step, placing one foot on the step followed by the other leg. Keep your knee positioned above your 2nd toe. Slowly step forward off the step. Lead with the step. Lead with the same leg to step on the step and off the step. (If difficulty stepping forward off the step then step back to the starting position and also	~12 RM	10-12 reps	2 sets	2 s concentric<br>1 s isometric<br>2 s eccentric	1 s between reps;<br>1 min between sets	50-60s/ set	**7A:** Step onto a low step (no risers), using light hand support as needed.<br>**7B:** Standing with your feet hit width apart, step forward with one leg while keeping body weight through the stationary leg.	**7A:** Step onto a medium step (1 riser), using light hand support as needed.<br>**7B:** Standing with your feet hit width apart, slide the one leg forward while keeping body weight through the stationary leg.	**7A:** Step onto a high step (2 risers), using light hand support as needed.<br>**7B:** If unable to do Level 3 7A, perform Level 1 7A.	**7A:** Step onto a high step (2 risers) holding a weight (load for 12RM) for up and down.<br>**7B:** If unable to do Level 3 7A, perform Level 2 7A.

	perform exercise 7B). Switch lead legs between sets.										
**8**<br>Pelvic lift/bridge<br>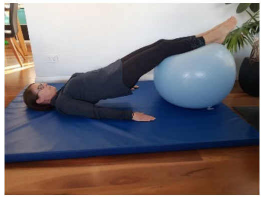	Lie on your back, feet hip width apart and calves resting on a gym ball. Lift your hips and buttocks off the floor towards the ceiling to straighten your hip. Slowly lower yourself back to the ground.	~12 RM	10-12 reps	2 sets	2 s concentric<br>1 s isometric<br>2 s eccentric	1 s between reps;<br>1 min between sets	50-60s/ set	Arms on the ground at your sides.	Arms across your chest.	Arms on the ground at your sides, one leg on the ball and the other in the air. Switch legs between sets.	Arms across your chest, one leg on the ball and the other leg in the air. Switch legs between sets. (Add weight plate across abdomen if needed.)
**9**<br>Balance<br>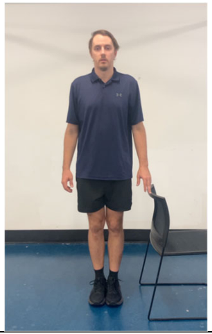	Stand with fingertip support as needed.	-	30 s	2 sets	-	1 min between sets	-	Stand with feet parallel, malleoli touching and equal weight on both legs.	Stand in modified tandem (medial tip of big toe touching medial heel).	Stand on one leg.	Standing on one leg and passing a ball around waist. (Additional: eyes closed)

Reps = repetitions; RM = repetition maximum, TUT = time under tension
